# Understanding the rural–urban disparity in acute respiratory infection symptoms among under-five children in Sub-Saharan Africa: a multivariate decomposition analysis

**DOI:** 10.1186/s12889-022-14421-0

**Published:** 2022-11-03

**Authors:** Getayeneh Antehunegn Tesema, Misganaw Gebrie Worku, Tesfa Sewunet Alamneh, Achamyeleh Birhanu Teshale, Yigizie Yeshaw, Adugnaw Zeleke Alem, Hiwotie Getaneh Ayalew, Alemneh Mekuriaw Liyew, Zemenu Tadesse Tessema

**Affiliations:** 1 Department of Epidemiology and Biostatistics, Institute of Public Health, College of Medicine and Health Sciences and Comprehensive Specialized Hospital, University of Gondar, Gondar, Ethiopia; 2 Department of Human Anatomy, College of Medicine and Health Sciences and Comprehensive Specialized Hospital, University of Gondar, Gondar, Ethiopia; 3 Department of Human Physiology, College of Medicine and Health Sciences and Comprehensive Specialized Hospital, University of Gondar, Gondar, Ethiopia; 4 Department of Midwifery, School of Nursing and Midwifery, College of Medicine and Health Sciences, Wollo University, Dessie, Ethiopia

**Keywords:** Acute respiratory infection symptoms, Under-five children, Sub-Saharan Africa

## Abstract

**Background:**

Acute Respiratory Infections (ARIs) account for more than 6% of the worldwide disease burden in children under the age of five, with the majority occurring in Sub-Saharan Africa. Rural children are more vulnerable to and disproportionately affected by ARIs. As a result, we examined the rural–urban disparity in the prevalence of ARI symptoms and associated factors among children under the age of five in Sub-Saharan Africa.

**Methods:**

We used the most recent Demographic and Health Survey (DHS) data from 36 countries in Sub-Saharan Africa. The study included 199,130 weighted samples in total. To identify variables associated with ARIs symptoms, a multilevel binary logistic regression model was fitted. The Adjusted Odds Ratio (AOR) with a 95% CI was used to determine the statistical significance and strength of the association. To explain the rural–urban disparity in ARI prevalence, a logit-based multivariate decomposition analysis was used.

**Results:**

Being female, ever breastfeeding, belonging to a poorer, better wealth status, and having better maternal educational status were significantly associated with lower odds of ARIs among under-five children. Whereas, small size or large size at birth, not taking vitamin A supplementation, being severely underweight, having diarrhea, didn’t have media exposure, never had the vaccination, being aged 36–47 months, and being aged 48–59 months were significantly associated with higher odds of ARIs among under-five children. The multivariate decomposition analysis revealed that the difference in characteristics (endowment) across residences explained 64.7% of the overall rural–urban difference in the prevalence of ARIs, while the difference in the effect of characteristics (change in coefficient) explained 35.3%.

**Conclusion:**

This study found that rural children were highly affected by ARIs in SSA. To reduce the excess ARIs in rural children, public health interventions aimed at impoverished households, home births, and unvaccinated and malnourished children are crucial.

## Background

Acute Respiratory Infections (ARIs) are a group of diseases caused by a wide range of pathogens such as Streptococcus pneumonia, Haemophilus influenza, Staphylococcus aureus, etc. [1]. ARIs are the major cause of illness and mortality in children under the age of five [2, 3]. They account for 6% of the global disease burden, which is greater than the burden of diarrheal illnesses and malaria combined [4–6]. Globally, ARIs are responsible for 12 million morbidities and 1.3 million fatalities in children under the age of five [7, 8], with three-fourths occurring in Sub-Saharan Africa (SSA) [9–18]. ARIs are responsible for the huge burden of infant and under-five mortality in rural areas [19].

According to studies, ARIs in children under the age of five are directly related to the population's environmental, cultural, and socioeconomic variables [20–23]. The prevalence of ARIs varied by residence, according to research undertaken in various Sub-Saharan African countries [24, 25], with rural households being the most afflicted due to the majority of people in rural areas relying predominantly on biomass for home energy [26]. Previous research found that maternal age, maternal education, place of delivery, Antenatal Care (ANC) visits, vitamin A supplementation, diarrhoea history, maternal age, household wealth status, child nutritional status, and other maternal-related factors have all been associated with ARIs symptoms in children under the age of five [22, 25, 27, 28].

Even though tremendous success in decreasing under-five mortality, there is significant variation among residents due to differences in susceptibility to common infectious diseases such as ARIs [29–31]. Even though various local studies on the prevalence and factors associated with ARIs among children under the age of five have been conducted in Sub-Saharan African countries [25, 32, 33], there is limited evidence on the rural–urban difference and factors that contributed to the change in ARIs across the residence. As a result, it's critical to investigate rural–urban disparity in ARIs among under-five children in SSA using multivariate decomposition analysis.

## Methods

### Data source and sampling procedure

This study used the Demographic and Health Surveys (DHSs) data of 36 sub-Saharan African countries conducted from 2005 to 2019, which was conducted using nationally representative samples to estimate core demographic and health indicators of the whole country. To recruit the samples, a multistage stratified cluster sampling technique was used, with Enumeration Areas (EAs) serving as primary sampling units and households serving as secondary sampling units [34]. The Kids Record dataset (KR file) was used for this study after we obtained an authorization letter from the measure DHS program for data access.

### Measurement of variables

#### Dependent variable

The outcome variable was ARI symptoms among under-five children. The presence of ARIs is defined as children having a history of cough within two weeks accompanied by short, rapid breathing or difficulty of breathing and fever within two weeks preceding the survey. In DHS, mothers of under-five children were asked whether their children had a history of cough within two weeks preceding the survey. For children who had a cough, the mother was asked whether the child's cough was accompanied by short, rapid breathing or difficulty of breathing and fever within two weeks preceding the survey. It was obtained from the question “did he/she breathe faster than usual with short, rapid breaths or have difficulty breathing in the 2 weeks preceding the survey?”. Then categorized as “Yes” if a child meets all the above-mentioned criteria and “No” otherwise [35].

#### Independent variables

The independent variables were categorized into child characteristics, mother characteristics, household characteristics, and contextual factors. Child characteristics include child age, sex of the child, breastfeeding, vitamin A supplementation, diarrhea in the last two weeks, ever had vaccinated, type of birth, child size at birth, and child nutritional status (stunting, wasting, and underweight); mothers-related characteristics include maternal age, media exposure, and maternal education, and household characteristics and contextual characteristics include household wealth status, residence, and country.

To assess a child's nutritional status DHS used anthropometric measures (height, age, and weight), height for age measures stunting, weight for height measured wasting, and weight for age measured for underweight. Stunting is defined as the height for age z-score less than 2 standard deviations below the median of the reference population, wasting is defined as the weight for height z-score less than 2 standard deviations below the median of the reference population, and underweight is defined as weight for age z-score less than 2 standard deviations below the median of reference population [36].

### Data analysis

To adjust for the non-response and sampling design, the data were weighted using the primary sampling unit, strata, and weighting variable. STATA version 16 statistical software was used for analysis. Since the DHS data has a hierarchical nature, under-five children within the same cluster might share similar characteristics to children from different clusters. This could violate the assumptions of the traditional logistic regression model; these are the independence of observations and equal variance assumptions. Therefore, a multilevel binary logistic regression model was fitted to identify factors associated with ARIs using EAs as a random variable. The presence of the clustering effect was assessed using the Intra-class Correlation Coefficient (ICC) and Likelihood Ratio (LR) test. ICC quantifies the degree of heterogeneity of ARIs between clusters (the proportion of the total observed individual-level variation in ARIs that is attributable to cluster variations)

$$\mathrm{ICC}={\sigma }^{2}/\left({\sigma }^{2}+\pi 2/3\right),$$

Six models were fitted and model comparison was made using deviance as the models were nested models. Null model (empty model), Model I (residence), Model II (Model I + child characteristics), Model III (Model II + mothers characteristics), Model IV (Model III + household characteristics), Model V (Model IV + country-level characteristics (sub-Saharan African region)) were fitted and a model with lowest deviance value was chosen as the best-fitted model for the data. We identified the independent variables based on previous literature conducted on determinants of ARIs. Variables with a *p*-value less than 0.2 in the bi-variable analysis were considered for multivariable analysis. The Adjusted Odds Ratio (AOR) with a 95% Confidence Interval (CI) and *p*-value < 0.05 in the multivariable model were used to declare significant determinants of ARIs.

Logit-based Multivariate Decomposition analysis was used to identify factors that contributed to the rural–urban difference in ARIs. The analysis was based on the logit link function which uses the output from the binary logistic regression model by dividing the difference in ARIs among under-five children into components. The overall rural–urban difference in the prevalence of ARIs among under-five children can be explained by the difference in composition between residences (i.e., differences in characteristics or endowment) and/or the difference in effects of the explanatory variables across residences (i.e., differences in coefficients). The mvdcmp STATA command was used to generate the overall and detailed multivariate decomposition analysis results [37]. Variables with a p-value < 0.2 in the bi-variable Logit-based multivariate decomposition analysis were considered for the multivariable Logit-based multivariate decomposition analysis. Finally, *p*-value < 0.05 and the corresponding coefficient (*B*) with a 95% confidence interval were used to declare significant factors that contributed to the rural–urban difference in ARIs.

### Ethical consideration

There was no need for ethical clearance as the researcher did not interact with respondents. The data used was obtained from the MEASURE DHS Program, and permission for data access was obtained from the measure DHS program through an online request from http://www.dhsprogram.com. The data used for this study were publicly available with no personal identifier. For details about the ethical considerations of the DHS, the program sees https://dhsprogram.com/methodology/Protecting-the-Privacy-of-DHS-Survey-Respondents.cfm.

## Results

### Background characteristics of respondents

The study included 199,130 children under the age of five. Around 135,832 (68.2%) of the under-fives were from rural areas. Nearly half (48.7%) of under-five children were of average birth weight, and about 96,264 (48.3%) were born to mothers aged 15–24 years (Table 1). Males composed more than half of under-five children in both rural (50.4%) and urban (50.31%) areas. About 19.47% of urban and 43.41% of rural under-five children were born to mothers with no formal education (Table 2).

**Table 1:** Distributions of acute respiratory infection symptoms across residence in sub-Saharan African countries (*n* = 199,330)

Countries	Study year	Number of under five children	Prevalence of ARI (95% CI)	Prevalence of ARI (95% CI)
			**Urban**	**Rural**
**Angola**	2015	12,528	3.3 (2.9, 3.7)	3.4 [2.9, 3.9]
**Burkina Faso**	2010	1407	18.9 [15.2, 23.2]	19.2 [16.9, 21.7]
**Benin**	2018	12,570	2.3 [1.9, 2.8]	3.2 [2.9, 3.7]
**Burundi**	2016/17	12,774	4.1 [3.1, 5.4]	7.4 [6.9, 7.9]
**Dr Congo**	2013/2014	5257	16.6 [15.0, 18.3]	24.4 [23.0, 25.9]
**Congo**	2011/2012	2129	16.5 [14.7, 18.6]	20.1 [17.4, 23.2]
**Cote di vaire**	2011/2012	1451	16.4 [13.6, 19.6]	17.2 [14.8, 19.9]
**Cameroon**	2011	3835	12.6 [11.2, 14.2]	17.3 [15.9, 19.1]
**Ethiopia**	2016	10,346	4.1 [3.1, 5.5]	7.0 [6.5, 7.5]
**Gabon**	2012	1860	21.1 [19.1, 23.2]	21.4 [17.0, 26.5]
**Ghana**	2014	748	17.4 [13.8, 21.7]	33.6 [29.0, 38.4]
**Gambia**	2013	1024	34.3 [30.3, 38.6]	33.7 [29.8, 37.9]
**Guinea**	2018	7152	1.9 [1.4, 2.6]	2.2 [1.9, 2.7]
**Kenya**	2014	6796	21.2 [19.6, 22.9]	24.3 [23.1, 25.6]
**Comoros**	2012/2013	545	14.3 [10.0, 20.1]	16.7 [13.2, 21.0]
**Liberia**	2019/2020	1508	24.8 [21.6, 28.2]	27.4 [24.5, 30.5]
**Lesotho**	2014	829	13.2 [9.5, 18.2]	17.5 [14.6, 20.7]
**Madagascar**	2008/09	1315	24.7 [19.3, 31.0]	26.5 [24.0, 29.2]
**Mali**	2018	9477	1.2 [0.8, 1.8]	2.2 [1.9, 2.6]
**Malawi**	2015/16	16,338	3.7 [3.0, 4.6]	5.7 [5.4, 6.1]
**Mozambique**	2011	1016	12.4 [9.5, 16.0]	18.9 [16.0, 22.2]
**Nigeria**	2018	30,779	2.0 [1.7, 2.2]	3.1 [2.8, 3.3]
**Niger**	2012	1735	24.7 [20.3, 30.0]	32.3 [29.9, 34.7]
**Namibia**	2013	1353	18.1 [15.3, 21.2]	20.8 [17.9, 24.0]
**Rwanda**	2014/15	2020	18.6 [14.9, 23.0]	21.8 [19.9, 23.8]
**Serra Leone**	2013	1988	17.3 [14.4, 20.6]	29.2 [26.9, 31.6]
**Senegal**	2010/2011	2242	28.4 [25.8, 31.2]	24.2 [21.8, 26.7]
**Sao tome**	2008/09	473	20.6 [15.6, 26.8]	43.1 [37.3, 49.1]
**Swaziland**	2005	679	39.0 [28.9, 50.2]	30.6 [27.0, 34.4]
**Chad**	2014/15	3279	33.3 [30.0, 36.7]	40.3 [38.4, 42.2]
**Togo**	2013/2014	1732	9.5 [7.5, 11.9]	14.6 [12.5, 16.8]
**Tanzania**	2015/16	9322	5.2 [4.4, 6.1]	3.3 [2.9, 3.8]
**Uganda**	2016	14,179	7.3 [6.4, 8.2]	10.2 [9.6, 10.7]
**South Africa**	2016	3289	3.5 [2.8, 4.4]	2.8 [2.1, 3.9]
**Zambia**	2018	9187	1.2 [0.9, 1.7]	2.0 [1.7, 2.4]
**Zimbabwe**	2015	5964	2.9 [2.2, 3.7]	4.3 [3.7, 5.0]

**Table 2:** Prevalence of ARIs and residential composition by study participants characteristics in SSA

Variables	Categories	ARI prevalence (%)	Residence	
			**Urban (%)**	**Rural(%)**
**Child’s sex**	Male	8.9	50.31	50.4
	Female	8.3	49.69	49.40
**Child’s age (months)**	< 12	9.7	21.56	22.02
	12–23	10.0	21.29	21.08
	24–35	8.8	19.91	19.19
	36–47	7.5	19.22	19.12
	48–59	6.5	18.02	18.59
**Child size at birth**	Average	6.9	49.77	48.14
	Small	11.4	16.25	19.32
	Large	9.5	33.98	32.53
**Maternal age (years)**	15–24	9.1	27.65	29.86
	25–34	8.5	51.29	46.97
	35–49	8.1	21.06	23.17
**Maternal education**	No formal	8.4	19.47	43.41
	Primary	9.6	27.53	39.67
	Secondary	7.8	43.15	15.68
	Higher	5.7	9.57	1.24
**Type of birth**	Single	8.6	96.85	97.05
	Twin	8.4	3.15	2.95
**Stunting**	Normal	8.4	43.94	37.87
	Moderate	8.0	9.60	13.17
	Severe	8.8	46.45	48.96
**Underweight**	Normal	8.2	51.58	48.44
	Moderate	8.7	5.07	8.04
	Severe	9.0	43.36	43.53
**Wasting**	Normal	8.2	55.48	61.13
	Moderate	10.8	2.18	2.80
	Severe	9.0	42.32	41.07
**Media exposure**	No	8.2	15.77	44.57
	Yes	8.8	84.23	53.43
**Wealth status**	Poorest	9.6	4.66	30.51
	Poorer	8.9	7.50	27.98
	Middle	8.5	15.20	22.24
	Richer	8.3	29.15	14.26
	Richest	7.2	43.51	5.00
**Vitamin A supplementation**	No	7.0	48.23	48.52
	Yes	10.1	51.77	51.48
**Breast feeding**	Ever breast feed	8.3	94.19	95.13
	Never breast feed	12.8	5.81	4.47
**Had diarrhea in the last 2 weeks**	No	7.0	82.49	80.78
	Yes	15.5	17.51	19.28
**Ever had vaccination**	No	4.6	39.58	44.46
	Yes	11.6	60.42	55.54
**Working status of mother**	No	8.3	36.77	33.69
	Yes	8.3	63.23	66.31

### Prevalence of ARIs across characteristics

In SSA, the overall prevalence of ARIs among children under the age of five was 8.6% (95% CI: 8.5%, 8.7%). The prevalence was 9.1% in rural areas and 7.5% in urban areas, respectively. ARIs were found in 9.7% of children aged less than 12 months and 10% of children aged 12 to 23 months. The chi-square test for the association of ARIs and independent variables showed a statistically significant association between residence, child sex, child age, child size at birth, maternal age, maternal education, stunting, underweight, wasting, media exposure, household wealth status, vitamin A supplementation, ever breastfeeding, ever had the vaccination had diarrhea in the last two weeks, and ARIs (*p*-value < 0.05) (Table 2).

### Factors associated with ARIs

Model V was chosen since it had the lowest deviance value. Residence, sex of the child, age of the child, size of child at birth, underweight, wasting, breastfeeding status, vitamin A supplementation, had diarrhea in the last two weeks, vaccination status, maternal education, media exposure, household wealth status, and sub-Saharan African region were found significant factors associated with ARI.

Being rural increased the odds of experiencing ARIs among under-five children by 1.25 times (AOR = 1.25, 95% CI: 1.20, 1.32) higher compared to urban under-five children. Children aged 36–47 months and 48–59 months were 1.29 times (AOR = 1.29, 95% CI: 1.21, 1.37) and 1.19 times (AOR = 1.19, 95% CI: 1.12, 1.26) higher odds of experiencing ARIs than those aged below 12 months, respectively. The odds of experiencing ARIs among female children were decreased by 5% (AOR = 0.95, 95% CI: 0.92, 0.98) compared to males. Being small size and large size at birth had 1.39 times (AOR = 1.39, 95% CI: 1.33, 1.45) and 1.28 times (AOR = 1.28, 95% CI: 1.23, 1.32) higher odds of experiencing ARIs compared to those normal-sized at birth. The odds of experiencing ARIs among children who breastfeed were decreased by 27% (AOR = 0.73, 95% CI: 0.66, 0.82) than those who never breastfeed their children. Children who did not take vitamin A had 45% (AOR = 1.45, 95% CI: 1.40, 1.50) higher odds of experiencing ARIs compared to those who took vitamin A.

Under-five children who didn't take childhood vaccination were 2.77 times (AOR = 2.77, 95% CI:2.64, 2.90) higher odds of experiencing ARIs compared to children who had a vaccination. The odds of experiencing ARIs among children who had a history of diarrhea were 1.98 (AOR = 1.98, 95% CI: 1.91, 2.05) times higher than those who didn't have a history of diarrhea. Children born to mothers who attained primary, secondary and higher education had 5% (AOR = 0.95, 95% CI: 0.91, 0.99), 26% (AOR = 0.74, 95% CI: 0.70, 0.78) and 46% (AOR = 0.54, 95% CI: 0.48, 0.62) decreased odds of experiencing ARIs compared to children born to mothers who did not have formal education, respectively. Children of mothers who did not have media exposure were 1.19 times (AOR = 1.19, 95% CI: 1.15, 1.24) had higher odds of experiencing ARIs than those born to mothers who had media exposure. The odds of experiencing ARIs among children belonged to poorer, middle, richer and richest households were decreased by 13% (AOR = 0.87, 95% CI: 0.83, 0.92), 16% (AOR = 0.84, 95% CI: 0.84, 0.89), 15% (AOR = 0.85, 95% CI: 0.80, 0.89) and 15% (AOR = 0.85, 95% CI: 0.79, 0.91) compared to those belonged to the poorest household quintile, respectively. The odds of experiencing ARIs among children in West Africa and Central Africa had 1.72 times (AOR = 1.72, 95% CI: 1.57, 1.88) and 2.16 times (AOR = 2.16, 95% CI: 2.06, 2.26) higher than those children in East Africa, respectively (Table 3).

**Table 3:** Factors associated with acute respiratory infection symptoms among under-five in sub-Saharan Africa

Variables	Null model	Model I (Residence)	Model II (Model I + child characteristics)	Model III (Model II + Mothers characteristics)	Model IV (Model III + Household characteristics)	Model V (Model IV + community-level characteristics)
**Residence**						
Urban		1	1	1	1	1
Rural		1.28 (1.24, 1.33)	1.30 (1.26, 1.35)	1.17 (1.12, 1.22)	1.11 (1.06, 1.16	1.25 (1.20, 1.32)^**^
**Sex of child**						
Male			1	1	1	1
Female			0.95 (0.92, 0.98)	0.95 (0.92, 0.98)	0.95 (0.92, 0.98)	0.95 (0.92, 0.98)^*^
**Child age (in months)**						
< 12			1	1	1	1
12–23			0.86 (0.82, 0.90)	0.85 (0.81, 0.89)	0.89 (0.85, 0.94)	0.84 (0.80, 1.03)
24–35			0.84 (0.80, 0.88)	0.83 (0.79, 0.87)	0.87 (0.83, 0.92)	0.83 (0.79, 1.01)
36–47			1.42 (1.34, 1.51)	1.44 (1.36, 1.53)	1.54 (1.46, 1.64)	1.29 (1.21, 1.37)^*^
48–59			1.32 (1.25, 1.41)	1.32 (1.24, 1.40)	1.42 (1.33, 1.51)	1.19 (1.12, 1.26)^*^
**Type of birth**						
Single			1	1	1	1
Multiple			0.90 (0.82, 0.99)	0.95 (0.86, 1.05)	0.95 (0.86, 1.05)	0.94 (0.85, 1.04)
**Child size at birth**						
Average			1	1	1	1
Small			1.57 (1.50, 1.64)	1.41 (1.35, 1.47)	1.41 (1.35, 1.47)	1.39 (1.33, 1.45)^**^
Large			1.35 (1.30, 1.40)	1.35 (1.30, 1.40)	1.35 (1.30, 1.40)	1.28 (1.23, 1.32)^**^
**Stunting**						
Normal			1	1	1	1
Moderate			0.93 (0.88, 0.98)	0.93 (0.87, 0.98)	0.92 (0.87, 1.04)	0.93 (0.87, 1.12)
Severe			0.89 (0.82, 0.95)	0.88 (0.81, 0.95)	0.87 (0.81, 0.99)	0.84 (0.78, 1.03)
**Underweight**						
Normal			1	1	1	1
Moderate			1.06 (0.98, 1.14)	1.04 (0.96, 1.12)	1.04 (0.97, 1.12)	1.04 (0.97, 1.25)
Severe			1.21 (1.08, 1.35)	1.19 (1.06, 1.33)	1.18 (1.05, 1.33)	1.19 (1.06, 1.34)^*^
**Wasting**						
Normal			1	1	1	1
Moderate			1.12 (1.02, 1.24)	1.14 (1.02, 1.26)	1.14 (1.03, 1.27)	1.13 (1.02, 1.26)^**^
Severe			1.06 (0.97, 1.17)	1.13 (1.02, 1.24)	1.13 (1.03, 1.25)	1.14 (1.04, 1.26)^**^
**Breastfeeding**						
Never breastfeed			1	1	1	1
Ever breastfeed			1.43 (1.34, 1.53)	0.81 (0.72, 0.90)	0.80 (0.72, 0.90)	0.73 (0.66, 0.82)^*^
**Vitamin A supplementation**						
Yes			1	1	1	1
No			1.26 (1.22, 1.30)	1.31 (1.26, 1.36)	1.31 (1.27, 1.36)	1.45 (1.40, 1.50)^**^
**Had diarrhea in the last two weeks**						
No			1	1	1	1
Yes			2.04 (1.97, 2.12)	2.04 (1.96, 2.11)	2.04 (1.97, 2.12)	1.98 (1.91, 2.05)^**^
**Ever had vaccination**						
Yes			1	1	1	1
No			3.13 (2.99, 3.28)	3.16 (3.01, 3.32)	3.33 (3.17, 3.49)	2.77 (2.64, 2.90)^*^
**Maternal educational status**						
No				1	1	1
Primary				1.05 (1.01, 1.09)	1.06 (1.02, 1.10)	0.95 (0.91, 0.99)^*^
Secondary				0.88 (0.84, 0.92	0.90 (0.86, 0.95)	0.74 (0.70, 0.78)^*^
Higher				0.57 (0.51, 0.65)	0.62 (0.54, 0.70)	0.54 (0.48, 0.62)^*^
**Place of delivery**						
Home				1	1	1
Health facility				0.80 (0.77, 0.83)	0.86 (0.82,0.89)	0.99 (0.94, 1.04)
**Working status**						
No				1	1	1
Yes				0.98 (0.95, 1.02)	0.98 (0.95, 1.02)	0.99 (0.95, 1.02)
**Media exposure**						
Yes					1	1
No					1.14 (1.10, 1.19)	1.19 (1.15, 1.24)^*^
**Household wealth status**						
Poorest					1	1
Poorer					0.87 (0.83, 0.91)	0.87 (0.83, 0.92)^*^
Middle					0.81 (0.77, 0.86)	0.84 (0.80, 0.89)^*^
Richer					0.78 (0.74, 0.82)	0.85 (0.80, 0.89)^*^
Richest					0.73 (0.68, 0.78)	0.85 (0.79, 0.91)^*^
**Sub-Saharan Africa region**						
East Africa						1
West Africa						1.72 (1.57, 1.88)^*^
Southern Africa						0.96 (0.92, 1.01)
Central Africa						2.16 (2.06, 2.26)^*^
LLR	-57,818/342	-57,726.622	-54,432.061	52,074.165	52,001.92	51,335.396
Deviance	115,636.684	115,453.244	108,864.122	104,148.330	104,003.84	102,670.792

### Decomposition of the rural–urban disparity ARI symptoms among under-five children in SSA

It was found that 64.7% of the overall rural–urban difference in ARIs prevalence was attributable to the difference in characteristics (endowment) across the residence and 35.3% was explained by the difference in the effect of characteristics (change in coefficient) across residences (Table 4). Among the endowment component, a female child (B = 0.033), child aged < 12 months (B = -0.0006), child aged 36–47 months (B = -0.0004), small-sized at birth (B = 0.0009), large size at birth (B = -0.0004), moderate wasting (B = 0.0006), severe wasting (B = 0.0004), having media exposure (B = -0.0003), having diarrhea (B = 0.0013), ever had vaccination (B = -0.0035), every breastfeeding (B = -0.0035), health facility delivery (B = -0.003), being in the poorest household (B = 0.009), poorer household (B = 0.005), middle household (B = 0.001), and richer household (B = -0.003) were significantly contributed for the change in ARIs prevalence across the residence.

**Table 4:** Overall decomposition analysis result of urban–rural disparity in acute respiratory infection symptoms among under-five children in sub-Saharan Africa

ARI	Coef. (95% CI)	Percent contribution
E	0.01 (0.009, 0.004)	64.7
C	0.006 (0.003, 0.01)	35.3
Residual	0.017 (0.015, 0.02)	

Among the coefficient components, a child aged less than 12 months (B = 0.0018), aged 36–47 months (B = 0.0014), ever had vaccination (B = -0.007), health facility delivery (B = -0.005), being from middle household wealth (B = 0.0013) and being from the richer household wealth index (B = 0.0025) were significant predictors contributed for the change in prevalence of ARIs across residence (Table 5).

**Table 5:** Results of decomposition of the rural–urban disparity in acute respiratory infection symptoms in under-five children in sub-Saharan Africa

Variables	Categories	Difference due to endowment			Difference due to coefficient		
		Coef with 95% CI	Pct	*p*-value	Coef with 95% CI	Pct	
**Sex of child**	Male	Ref			Ref		
	Female	0.033 (0.071, 0.59)^*^	0.02	0.001	0.0003 (-0.00017, 0.0019)	1.98	0.41
**Type of birth**	Single	Ref			Ref		
	Twin	0.0006 (-0.26, 0.0001)	0.04	0.161	-0.0002 (-0.004, 0.001)	-0.97	0.76
**Child age (months)**	< 12	-0.0006 (-0.0007, -0.0004)^*^	-0.33	0.021	0.0018 (0.0006, 0.003)^*^	10.5	0.02
	12–23	0.0004 (0.0003, 0.0005)^*^	0.24	0.031	0.0014 (0.00017, 0.0026)^*^	8.1	0.03
	24–35	0.0002 (0.0001, 0.0003)^*^	1.1	0.001	0.001 (-0.0005, 0.0023)	6.4	0.101
	36–47	-0.0004 (-0.0008, -0.002)^*^	-0.02	0.035	0.0006 (-0.001, 0.001)	0.34	0.2
	48–59	Ref			Ref		
**Child size at birth**	Average	Ref			Ref		
	Small	0.0009 (0.0007, 0.001)^*^	5.15	0.041	-0.0007 (-0.001, 0.0003)	-3.7	0.132
	Large	-0.0004 (-0.0005, -0.003)^*^	-2.33	0.02	-0.0019 (-0.001, 0.001)	-1.11	0.432
**Wasting**	Normal	Ref			Ref		
	Moderate	0.0006 (0.00006, 0.0001)^*^	0.32	0.04	-0.0001 (-0.001, 0.0008)	-0.7	0.42
	Severe	0.0004 (0.0003, 0.0005)^*^	0.23	0.032	0.0013 (-0.007, 0.003)	7.5	0.34
**Media exposure**	No	Ref			Ref		
	Yes	-0.003 (-0.004, -0.002)^*^	16.6	0.012	-0.0005 (-0.004, 0.003)	3.15	0.61
**Diarrhea in the last two weeks**	No	Ref			Ref		
	Yes	0.0013 (0.0012, 0.0014)*	7.5	0.034	-0.0001 (-0.0007, 0.0005)	-0.69	0.31
**Ever breast feeding**	No	Ref			Ref		
	Yes	-0.0024 (-0.0030, -0.0018)^*^	-1.38	0.04	0.0006 (-0.0003, 0.0004)	0.34	0.43
**Ever had vaccination**	No	Ref			Ref		
	Yes	-0.0035 (-0.0038, -0.0033)^*^	-20.26	0.023	-0.007 (-0.009, -0.005)^*^	-40.9	0.0023
**Place of delivery**	Home	Ref			Ref		
	Health facility	-0.003 (-0.004, -0.002)^*^	18.14	0.03	-0.005 (-0.008, -0.001)	-26.6	0.003
**Wealth status**	Poorest	0.009 (0.008, 0.011)^*^	54.42	0.01	-0.0006 (-0.001, -0.00008)^*^	1.16	0.021
	Poorer	0.005 (0.003, 0.006)^*^	27.39	0.042	0.0002 (-0.0004, 0.0008)	1.16	0.12
	Middle	0.001 (0.0007, 0.0014)^*^	6.11	0.028	0.0013(0.0003, 0.002)^*^	7.26	0.02
	Richer	-0.003 (-0.004, -0.0015)^*^	-15.17	0.019	0.0025 (0.001, 0.004)*	14.6	0.031
	Richest	Ref			Ref		

## Discussion

This study found a higher prevalence of ARIs among under-five children in rural areas. In addition, in the multilevel analysis, the odds of experiencing ARIs among under-five children in rural areas were higher than in under-five children in urban areas. The possible explanation for the rural–urban disparity in ARIs among under-five children might be due to limited health care service availability and accessibility in rural areas of LMICs specifically in sub-Saharan African countries, responsible for the large concentration of acute respiratory tract infections in under-five children in rural areas [38, 39]. Besides, the literature showed that under-five children in rural households are more likely suffered from undernutrition, which in turn, increases their susceptibility to childhood infections such as pneumonia, bronchiolitis, tosilo-pharyngitis, and otitis media [40, 41]. Moreover, a majority of children in rural areas of sub-Saharan African countries do not receive full vaccinations against common childhood illnesses [42–44] and have higher exposure to household air pollution from unprocessed fuel, which is identified as the substantial cause of respiratory illness among under-five children [45].

Female children had lower odds of experiencing ARIs compared to male children. This finding is in line with previous studies reported in Egypt [16] and Bangladesh [14]. The possible explanation might be due to sex differences in genetic and biological makeup, with a male child being biologically weaker and more susceptible to respiratory diseases [46].

Another important factor significantly associated with ARIs was child age. A child aged 37–47 months and 48—59 months had higher odds of experiencing ARIs compared to children aged less than 12 months. It is consistent with studies reported in developing countries [47, 48], this could be due to infants being relatively at a decreased level of exposure to different types of infections related to eating contaminated food prepared and limited exposure to the unhealthy environment compared to older children [49, 50]. On contrary, older children are more exposed to the environmental risk factors of ARIs as they child walk and have contact with different individuals [51].

Being small or large size at birth was significantly associated with higher odds of ARIs compared to an average-sized child at birth. This is consistent with study findings in India [52] and Argentina [53]. These might be due to small and large size children at birth having commonly underlined medical conditions like malnutrition, chronic illnesses such as down syndrome, congenital heart diseases, and infectious diseases, in turn, these increase their risk of infections [54, 55]. The odds of experiencing ARIs among children who didn’t take vitamin A supplementation were lower compared to children who took vitamin A supplementation. It is supported by a previous study [56], this might be because vitamin A plays a vital role in the development of the epithelium mucosae of the respiratory tract and improves humoral and cellular immunity by influencing the synthesis of immunoglobulins [57].

Having diarrhea in the last two weeks increases the odds of experiencing ARIs compared to children who did not have a history of diarrhea. This is consistent with study findings in Indonesia [58], and Bangladesh [59], it could be due to diarrhea can cause nutritional depletion because of the loss of zinc and other nutrients, which in turn increases the risk of ARIs [60]. Unvaccinated children had lower odds of experiencing ARIs compared to vaccinated children, which is supported by literature reported in India [61], and Cameroon [23]. Common childhood vaccines like PCV and pentavalent are effective in preventing common respiratory infections caused by streptococcus pneumonia, H-influenza, and measles, and therefore unvaccinated children are at higher risk of contracting ARIs [62].

Maternal education and wealth status were found to be significant predictors of ARIs. Better wealth and higher maternal education are responsible for decreased risk of ARIs among under-five children. These are supported by studies reported in Bangladesh [63], Iraq [28], and Afghanistan [64]. The possible explanation might be due to education enhancing mother's healthcare-seeking behavior and their level of adherence to appropriate childcare practices like exclusively breastfeeding children until 6 months, initiation of appropriate complementary feeding, and vaccinating their children, which could, in turn, reduce the risk of ARIs [65, 66]. Health facility delivery lowers the odds of ARIs among under-five children, it is supported by previous studies [48, 67]. Under-five children born to mothers who had no media exposure had higher odds of experiencing ARIs than those children born to mothers who didn't have media exposure. Media exposure enhances the knowledge level of women to be able to use maternal health care services such as ANC, health facility delivery, PNC, appropriate child feeding practices, and child vaccinations, which could reduce their risk of infections [14].

In the multivariate decomposition analysis, the urban–rural disparity in ARIs among under-five children was due to the difference in the composition of sex of the child, child age, child size at birth, wasting status, media exposure, having a diarrheal illness, ever had the vaccination, ever breastfeeding, health facility delivery and household wealth status, and change in the effect of child age, ever had the vaccination, health facility delivery and household wealth status across the residence. It could be due to improved wealth, health facility delivery, breastfeeding, childhood vaccination, and media exposure can improve childhood nutritional status, which in turn improves immune competency [68, 69]. Several childhood vaccines against respiratory infections are currently available and are part of the Expanded Program of Immunization (EPI) schedule [70, 71]. Therefore, the decreased incidence of ARIs in urban areas might be attributable to the increased immunization coverage in urban areas specifically in SSA [72]. In addition, breast milk provides immunologic protection against numerous illnesses during childhood, therefore breastfeeding children rather than formula-feeding children has decreased the incidence of acute respiratory tract infections [73, 74]. Overall, these could be responsible for the decreased incidence of ARIs among under-five children in urban areas.

### Policy implications

In this study, we found that rural children under five were highly affected by ARIs. This information has been used as a preventive measure that is linked to maternal and child health. From a policy point of view, the interventions which are designed to tackle ARIs such as childhood vaccination, nutrition, environmental sanitation, exclusive breastfeeding practice, and maternal education should be scaled to sustain the reduction in ARIs related to under-five mortality in SSA. Childhood vaccination and nutrition are vital to reducing childhood mortality and morbidity. Enhancing the availability of education to women is needed to increase the chance of child survival as they adhered to maternal and child health guidelines and recommendations.

### Strengths and limitations of the study

The study was done based on the weighted Demographic and Health Survey (DHS) data of 36 SSA to ensure representativeness and to obtain reliable estimates. As the study was cross-sectional, we are unable to show a temporal relationship; however, multilevel modeling was employed to consider the clustering effect to obtain reliable estimates and SE. In addition, all information related to ARIs was provided by mothers and was not validated by applying medical examinations/investigation, and was thus subjective. Therefore, it is prone to recall bias since the retrospective data on their previous history were collected.

## Conclusion

Under-five children in rural areas were more likely to suffer from ARIs in SSA. Public health interventions should target children in poor households, and unvaccinated and undernourished children to narrow the rural–urban disparity in ARIs. Sex of children, child age, child size at birth, vitamin A supplementation, diarrhea in the last two weeks, ever had the vaccination, maternal education, wealth status, media exposure, and breastfeeding status were significantly associated with ARIs in the multivariable analysis. Therefore, enhancing maternal education, health facility delivery, vitamin A supplementation, and childhood vaccination is vital to reduce the incidence of ARIs among under-five children.

## Data Availability

Data is available online and you can access it from www.measuredhs.com.

## Abbreviations

AOR: Adjusted odds ratio
ARIs: Acute respiratory infection symptoms
CI: Confidence interval
CPR: DHS: Demographic and health survey
ICC: Intra-class correlation coefficient
LLR: Log-likelihood Ratio
LR: Likelihood ratio
SSA: Sub-Saharan Africa
WHO: World health organization
